# Cardiorespiratory Fitness, Coronary Artery Calcium, and Risk of Later-Life Dementia: The Cooper Center Longitudinal Study

**DOI:** 10.1016/j.jacadv.2026.103079

**Published:** 2026-08-07

**Authors:** Yariv Gerber, David Leonard, Kerem Shuval, Carolyn E. Barlow, Clare Meernik, Andjelka Pavlovic, Qing Li, Duke Appiah, Kelley Pettee Gabriel, Nina B. Radford, Laura F. DeFina

**Affiliations:** a Department of Epidemiology and Preventive Medicine, School of Public Health, Gray Faculty of Medical & Health Sciences, Tel Aviv University, Tel Aviv, Israel; b Kenneth H. Cooper Institute, Texas Tech University Health Sciences Center, Dallas, Texas, USA; c Julia Jones Matthews School of Population and Public Health, Texas Tech University Health Sciences Center, Lubbock, Texas, USA; d Department of Epidemiology, University of Alabama at Birmingham, Birmingham, Alabama, USA; e Cooper Clinic, Dallas, Texas, USA

**Keywords:** brain health, cardiorespiratory fitness, cohort study, coronary artery calcium, dementia, subclinical atherosclerosis

## Abstract

**Background:**

Cardiorespiratory fitness (CRF) and coronary artery calcium (CAC) are established cardiovascular risk markers reflecting physiological reserve and subclinical atherosclerosis, respectively. Their independent and joint associations with dementia remain unclear.

**Objectives:**

We examined mid-to-late midlife CRF and CAC in relation to later-life dementia within a large U.S. cohort.

**Methods:**

Participants in the Cooper Center Longitudinal Study (1998-2018) were linked to Medicare claims (1999-2019). CRF was estimated via maximal treadmill testing (categorized as age- and sex-specific low, moderate, or high); CAC was categorized as <100 vs ≥100 Agatston units. Incident all-cause dementia was identified via Medicare surveillance. Parametric illness-death models estimated HRs for dementia, accounting for competing mortality and adjusting for demographic, behavioral, and cardiometabolic risk factors, with mutual adjustment for CRF and CAC.

**Results:**

Among 10,933 participants free of cardiovascular disease and dementia at baseline (mean age 57.2 years; 30.2% women), 868 dementia cases occurred over a median follow-up of 5.8 years. After multivariable adjustment, high CRF (vs low) was associated with a lower dementia risk (HR: 0.69; 95% CI: 0.53-0.90), whereas CAC ≥100 (vs <100) was associated with a higher risk (HR: 1.24; 95% CI: 1.07-1.44). Continuous analyses demonstrated a linear inverse association for CRF and a graded positive association for CAC. No interaction between CRF and CAC was observed.

**Conclusions:**

Lower CRF and higher CAC are independently associated with incident dementia. These findings support complementary, nonoverlapping roles of functional fitness and cumulative vascular injury in dementia pathogenesis, highlighting the importance of lifelong cardiovascular health.

Dementia is a major public health concern, with a growing burden as populations age and with limited disease-modifying treatments available.^1^^,^^2^ The number of people living with dementia worldwide is projected to increase from approximately 57 million in 2019 to more than 150 million by 2050.^3^ Accumulating evidence indicates that the pathological processes leading to dementia begin decades before clinical onset,^4^, ^5^, ^6^ highlighting midlife as a critical window for risk identification and prevention.^6^, ^7^, ^8^ Cardiovascular health, in particular, has emerged as a central determinant of brain aging, with vascular and lifestyle-related factors implicated in both cognitive decline and dementia.^8^, ^9^, ^10^, ^11^ Identifying midlife cardiovascular disease (CVD) markers that capture long-term risk and are potentially modifiable is of major relevance.^7^^,^^12^

Cardiorespiratory fitness (CRF), an objective physiological measure closely linked to habitual physical activity, and coronary artery calcium (CAC), a quantitative, noninvasive marker of subclinical atherosclerosis, are widely used in CVD risk assessment.^13^, ^14^, ^15^, ^16^ Yet, their relevance to brain aging remains incompletely understood. Although CRF has been associated with a lower dementia risk,^17^, ^18^, ^19^, ^20^, ^21^ evidence linking CAC to cognitive outcomes is limited and largely preliminary,^22^^,^^23^ and whether it contributes directly to cognitive decline or merely signals shared risk profiles is still unclear.^24^ These 2 markers reflect distinct yet potentially related physiological processes: cardiovascular, pulmonary, and muscular reserve in the case of CRF and subclinical atherosclerosis in the case of CAC. They may interact biologically,^13^^,^^14^^,^^25^, ^26^, ^27^ influencing dementia risk through overlapping vascular and nonvascular mechanisms.

The Cooper Center Longitudinal Study (CCLS), a large, well-characterized cohort of generally healthy adults, offers a unique opportunity to evaluate the independent and combined associations of CRF and CAC, assessed in predominantly middle-aged adults, with dementia risk in later life.^13^^,^^16^^,^^17^ Leveraging long-term dementia follow-up through linkage of the CCLS to Medicare data allowed us to examine whether lower CRF and higher CAC were independently associated with dementia risk and whether the association between CAC and dementia varied by CRF.

## Methods

### Study population

The CCLS is a prospective cohort of community-dwelling adults who undergo preventive health evaluations at the Cooper Clinic in Dallas, Texas. It aims to assess the association between lifestyle behaviors and morbidity and mortality.^13^^,^^16^^,^^17^ Participants are typically self-referred or referred by their employers and complete standardized examinations that include clinical assessments, laboratory testing, and a maximal treadmill exercise test to assess CRF. They were predominantly of White race, well educated, and had access to medical care.^16^ All participants provided written informed consent to take part in the study, and the CCLS received ethics approval from the Texas Tech University Health Sciences Center Institutional Review Board (IRB-FY2025-106).

**Figure 1:**
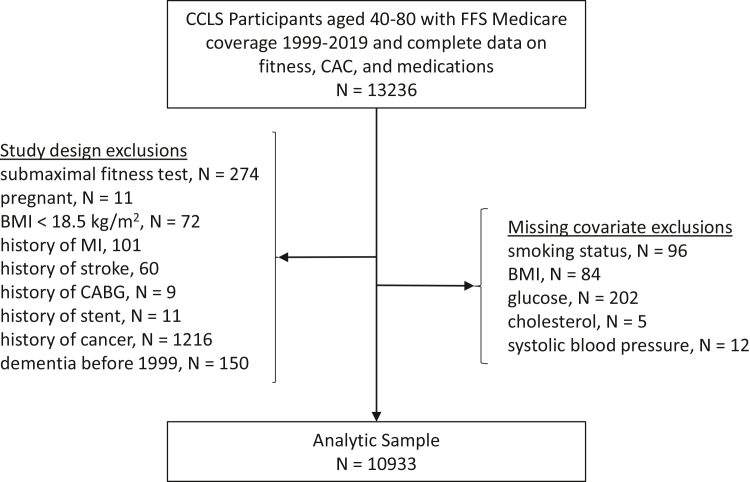
**Flow Diagram of Study Population Selection** Flow diagram of participant selection for the analytic cohort. The initial sample included 13,236 CCLS participants aged 40 to 80 years with fee-for-service Medicare coverage (1999-2019) and complete data on fitness, coronary artery calcium (CAC), and medications. Participants were excluded based on predefined study design criteria and missing covariate data. The final analytic sample consisted of 10,933 individuals. BMI = body mass index; CABG = coronary artery bypass grafting; CCLS = Cooper Center Longitudinal Study; FFS = fee-for-service; MI = myocardial infarction.

For this analysis, we included CCLS participants aged 40 to 80 years who underwent a treadmill fitness test and a CAC scan between 1998 and 2018. These participants were linked to Medicare claims data from 1999 to 2019, allowing for outcome surveillance. We excluded individuals with a history of myocardial infarction, stroke, coronary revascularization procedures, cancer (excluding nonmelanoma skin cancer), or dementia before the start of Medicare surveillance. Additional exclusions included pregnancy, underweight status (body mass index [BMI] <18.5 kg/m^2^), submaximal effort on treadmill testing (defined as achieving <85% of age-predicted maximal heart rate), and missing data on key covariates. The final analytic sample was 10,933 individuals (Figure 1).

### Assessment of cardiorespiratory fitness

CRF was objectively assessed using a physician or symptom-limited maximal treadmill exercise test. Testing followed a modified Balke protocol, a standardized and well-validated method used in CCLS.^13^^,^^17^^,^^21^^,^^28^ CRF was expressed in metabolic equivalents (METs), with 1 MET defined as 3.5 mL O_2_/kg/min. METs were estimated from the final treadmill speed and grade using standard regression equations.^29^ This method is highly correlated with directly measured maximal oxygen uptake (VO_2_max).^30^^,^^31^ We assigned participants to age- and sex-specific CRF categories (low, moderate, or high) based on their estimated MET values. These categories were derived from previously established CCLS cutoffs and reflect thresholds associated with clinical outcomes.^13^^,^^17^^,^^21^^,^^28^ Leisure-time physical activity data were available for 9,788 participants (89.5%) in the analytic sample. Approximately one-fifth reported no regular exercise, whereas nearly one-half reported participation in at least 1 vigorous-intensity activity, including running, cycling, or swimming.

### Assessment of coronary artery calcium

CAC was assessed using noncontrast cardiac computed tomography. From 1998 to 2007, CAC was measured using electron beam tomography with the C-150XP or C-300 systems (GE Imatron). From 2008 to 2018, a 64-slice multidetector computed tomography scanner (Lightspeed VCT; GE Healthcare) was used.^13^^,^^16^^,^^32^ All scans were acquired using a standard breath-hold protocol with a slice thickness of 3 mm and a table increment of 2 mm. CAC scores were computed using the Agatston method, which incorporates lesion area and peak attenuation.^33^ The scoring methods used in this cohort have been previously validated and shown to be highly reproducible and free of systematic bias.^13^^,^^32^ In the primary analysis, CAC was dichotomized as <100 or ≥100 AU to differentiate participants with minimal or no calcification from those with a clinically relevant burden of subclinical coronary atherosclerosis, which is associated with increased CVD risk.^32^

### Other covariates

Details of the clinical evaluation have been previously described.^13^^,^^16^^,^^17^^,^^28^^,^^32^ Participants reported demographic characteristics, including age, sex, race and ethnicity, and educational attainment (college graduate or higher vs less than college). The year of examination was included as a covariate to partly account for secular trends in exposures and ascertainment. Clinical measurements included BMI calculated from measured height and weight, and resting blood pressure obtained using standard protocols. Fasting blood samples were analyzed for plasma glucose and total cholesterol. Current smoking status was self-reported, as was the use of antihypertensive and lipid-lowering medications. In survival models, stroke or transient ischemic attack (TIA), identified from Centers for Medicare & Medicaid Services (CMS) claims, was included as a time-dependent interim event. Apolipoprotein E (ApoE) genotype (ε4 carrier status), a major genetic risk factor for Alzheimer disease,^6^^,^^34^ was measured in a subsample (n = 1,790) who participated in the 2008 to 2013 CI-UTSW BioBank, independent of study outcomes.

### Outcome ascertainment: dementia from Medicare claims

Incident all-cause dementia was identified through linkage with Medicare administrative claims data during the follow-up period from 1999 to 2019, using the CMS Chronic Conditions Warehouse algorithm.^17^^,^^21^ This algorithm applies a 3-year reference period, during which participants must have at least 1 qualifying claim from an inpatient hospital, skilled nursing facility, home health agency, hospital outpatient department, or physician/supplier service. Qualifying diagnoses were based on the International Classification of Diseases (ICD) system. For records before October 2015, the following ICD-9-Clinical Modification codes were used: 331.0, 331.1, 331.11, 331.19, 331.2, 331.7, 290.0, 290.10 to 290.13, 290.20, 290.21, 290.3, 290.40 to 290.43, 294.0, 294.10, 294.11, 294.20, 294.21, 294.8, and 797. For claims from October 2015 onward, the following ICD-10-Clinical Modification codes were applied: F01.50, F01.51, F02.80, F02.81, F03.90, F03.91, F04, G13.8, F05, F06.1, F06.8, G30.0, G30.1, G30.8, G30.9, G31.1, G31.2, G31.01, G31.09, G94, R41.81, and R54. The date of the first qualifying claim was used to define the timing of dementia onset.^17^^,^^21^ This approach, widely used in epidemiologic studies of aging and cognitive outcomes, has demonstrated moderate to high validity, often showing high specificity with somewhat lower sensitivity in older adult populations.^35^ An additional outcome was all-cause mortality, which served as a competing event. Data on all-cause mortality were available from CMS claims files.^16^

### Statistical analysis

Clinical characteristics of participants at baseline were summarized across CRF and CAC categories. We used parametric illness-death models with semicompeting risks to jointly model time to incident all-cause dementia and death without prior dementia, recognizing that death precludes the subsequent observation of dementia.^16^^,^^21^ We reported HRs and 95% CIs for dementia, our primary outcome. Models were estimated using the NLMIXED procedure in SAS, specifying Gompertz baseline hazard functions of age stratified by sex, which allowed the baseline risk to differ between men and women. Age at the clinic examination, when CRF and CAC were assessed, was also included as a covariate. Thus, comparisons were made among individuals of similar attained age during follow-up while accounting for differences in the age at which exposures were measured. A (unit mean) gamma-distributed shared frailty term was included to account for unobserved heterogeneity across individuals.^36^ The primary exposures were CRF and CAC, as assessed at the clinic visit; CRF was categorized into 3 levels (low, moderate, and high), and CAC was dichotomized (<100 vs ≥100 AU). In sensitivity analysis, CAC was classified into the standard categories 0, 1 to 99, 100 to 399, 400 to 999, and ≥1,000 AU. Nonlinear dementia hazards were modeled by expanding continuous CRF and $\sqrt{\text{CAC}}$ in Chebyshev polynomials up to degree 5.^37^ Models were sequentially (cumulatively) adjusted for the following covariates, selected a priori: age at the clinic examination, exam year, education level, BMI, and current smoking status (with baseline hazards stratified by sex; model 1); fasting glucose, total cholesterol, and systolic blood pressure (model 2); and both CRF and CAC (model 3). Additional adjustment for antihypertensive and statin therapy (model 4) and for stroke or TIA as a time-dependent covariate (model 5) was performed as sensitivity analyses. Potential effect modification was evaluated by including multiplicative interaction terms (CRF × CAC). ApoE variant frequencies in a subgroup of n = 1790 with this information were summarized by CRF and CAC and tested using rank-sum statistics. All statistical tests were 2-sided, and *P* values <0.05 were considered statistically significant. Analyses were performed using SAS (version 9.4; SAS Institute).

## Results

Among 10,933 participants free of CVD and dementia at baseline (mean [SD] age, 57.2 [6.9] years; 30.2% women), 28.0% had CAC ≥100 AU; the mean CRF was 10.1 METs at maximal workload, and cardiometabolic profiles were generally favorable. Apparent and graded differences in clinical characteristics were observed across CRF categories. On average, participants with higher CRF had lower BMI, blood pressure, and fasting glucose, more favorable lipid profiles, and a substantially lower prevalence of current smoking. Physical activity levels increased markedly across higher CRF categories. The prevalence of CAC ≥100 AU decreased across CRF categories, from 31.9% in the low CRF group to 29.1% in the moderate group and 26.8% in the high CRF group. The use of antihypertensive and statin medications was also progressively lower among participants with higher CRF. In contrast, differences between CAC categories reflected distinct risk profiles. Participants with CAC ≥100 AU were older, predominantly men, and had higher blood pressure and fasting glucose than those with CAC <100 AU. They were also more likely to be treated with antihypertensive and statin medications (Table 1). The Spearman correlation between CAC and CRF was weakly inverse (r = −0.16 in men and −0.15 in women) and attenuated to near null after age adjustment (r = −0.03 in both sexes).

**Table 1:** Clinical Characteristics of CCLS Participants According to Categories of Cardiorespiratory Fitness and Coronary Artery Calcium

Characteristic	All (N = 10,933, 100.0%)	CRF			CAC	
		Low (n = 9069, 803%)	Moderate (n = 3,810, 34.8%)	High (n = 6,214, 56.8%)	<100 AU (n = 7,871, 72.0%)	≥100 AU (n = 3,062, 28.0)
Age at baseline, mean (SD), y	57.2 (6.9)	56.7 (7.4)	57.4 (7.3)	57.2 (6.5)	55.9 (6.3)	60.7 (7.1)
Men, n (%)	7,635 (69.8)	668 (73.5)	2,845 (74.7)	4,122 (66.3)	4,893 (62.2)	2,742 (89.5)
White,^a^ n (%)	10,500 (96.0)	837 (92.1)	3,638 (95.5)	6,025 (97.0)	7,528 (95.6)	2,972 (97.1)
College graduate,^a^ n (%)	7,624 (69.7)	552 (60.7)	2,496 (65.5)	4,576 (73.6)	5,447 (69.2)	2,177 (71.1)
Current smoker, n (%)	1,075 (9.8)	159 (17.5)	461 (12.1)	455 (7.3)	718 (9.1)	357 (11.7)
Alcohol use, mean (SD), drinks/week	6.2 (7.1)	5.2 (7.2)	6.1 (7.5)	6.4 (6.8)	5.9 (6.8)	7.1 (7.9)
PA,^a^ mean (SD), MET-minutes/week	1,083.4 (1,336.3)	390.3 (717.9)	688.7 (1,076.5)	1,432.7 (1,440.5)	1,056.5 (1,343.4)	1,155.9 (1,314.5)
CRF, mean (SD), METs	10.1 (2.1)	7.1 (1.2)	9 (1.4)	11.2 (1.8)	10.1 (2.1)	10.1 (2.1)
CAC, mean (SD), AU	200.8 (564.8)	245 (593.8)	212.2 (599.8)	187.4 (537.4)	12.4 (23.8)	685.2 (901.1)
BMI, mean (SD), kg/m^2^	27 (4.3)	32 (5.5)	28.4 (4.0)	25.4 (3.3)	26.7 (4.3)	27.7 (4.2)
Total cholesterol, mean (SD), mg/dL	201.5 (37.3)	203.5 (40.9)	202.8 (38.9)	200.5 (35.7)	203.4 (36.2)	196.7 (39.5)
LDL cholesterol,^a^ mean (SD), mg/dL	121.2 (32.7)	123.9 (33.7)	123.3 (33.8)	119.5 (31.7)	122 (32.1)	119.1 (33.9)
HDL cholesterol,^a^ mean (SD), mg/dL	55.8 (17.2)	48.3 (15.0)	51.6 (15.7)	59.5 (17.4)	57.3 (17.7)	52.0 (15.0)
Triglycerides,^a^ mean (SD), mg/dL	125.2 (90.3)	162.9 (127.1)	143 (98.6)	108.7 (73.2)	122.9 (84.5)	131 (103.5)
Glucose, mean (SD), mg/dL	99.3 (17.4)	107 (26.1)	101.5 (20.7)	96.8 (12.5)	98.2 (16.5)	102.1 (19.4)
Systolic BP, mean (SD), mmHg	125.0 (15.4)	129.3 (15.9)	126.5 (15.3)	123.5 (15.1)	123.4 (14.9)	129.3 (15.6)
Diastolic BP, mean (SD), mmHg	82.6 (9.7)	85.4 (10.6)	83.8 (9.8)	81.5 (9.4)	82.2 (9.7)	83.6 (9.7)
Antihypertensive medication, n (%)	2,371 (21.7)	301 (33.1)	1,000 (26.2)	1,070 (17.2)	1,371 (17.4)	1,000 (32.7)
Statin medication, n (%)	2063 (18.9)	196 (21.6)	791 (20.8)	1,076 (17.3)	1,033 (13.1)	1,030 (33.6)

BMI = body mass index; BP = blood pressure; CAC = coronary artery calcium; CCLS = Cooper Center Longitudinal Study; CRF = cardiorespiratory fitness; HDL = high-density lipoprotein; LDL = low-density lipoprotein; MET = metabolic equivalent of task; MI = myocardial infarction; PA = physical activity.

a Number of participants with missing data: race or ethnicity (n = 15); college education (n = 370); alcohol consumption (n = 898); physical activity (n = 1,145); LDL cholesterol (n = 131); HDL cholesterol (n = 3); and triglycerides (n = 1).

### Associations of CRF and CAC with dementia

During Medicare follow-up, which began a median (Q1, Q3) of 8.9 (3.8, 13.2) years after the clinic visit and lasted a median (Q1, Q3) of 5.8 (2.5, 10.0) years, 868 participants developed all-cause dementia (mean [SD] age at diagnosis, 76.5 [6.8] years). Dementia incidence rates (per 1,000 person-years) declined across increasing categories of CRF, from 14.8 among those with low fitness, to 13.2 among those with moderate fitness, and 10.8 among those with high fitness. Rates were substantially higher among participants with CAC ≥100 AU compared with those with CAC <100 AU (15.9 vs 9.7) (Table 2). Classifying CAC scores into more refined categories, dementia rates per 1,000 person-years (95% CIs) were 9.0 (8.0-10.2) for no CAC (0 AU; n = 4,912), 10.7 (9.3-12.2) for 1 to 99 AU (n = 2,959), 13.0 (11.1-15.1) for 100 to 399 AU (n = 1,667), 18.9 (16.0-22.4) for 400 to 999 AU (n = 798), and 18.6 (15.4-22.4) for ≥1,000 AU (n = 597).

**Table 2:** All-Cause Dementia HRs (95% CIs) for Cardiorespiratory Fitness and Coronary Artery Calcium in 10,933 Participants of the Cooper Center Longitudinal Study With Medicare Follow-Up From 1999-2019

Analysis	CRF Categories			CAC Categories	
	Low (n = 909, 8.3%)	Moderate (n = 3,810, 34.8%)	High (n = 6,214, 56.8%)	<100 AU (n = 7,871, 72.0%)	≥100 AU (n = 3,062, 28.0%)
Number of events	74	321	473	460	408
Incidence rate (95% CI)^f^	14.8 (11.8-18.6)	13.2 (11.9-14.8)	10.8 (9.9-11.8)	9.7 (8.9-10.6)	15.9 (14.4-17.5)
HRs (95% CI)					
Model 1^a^	1 (ref)	0.86 (0.67-1.12)	0.69 (0.53-0.90)	1 (ref)	1.24 (1.07-1.45)
Model 2^b^	1 (ref)	0.86 (0.66-1.11)	0.68 (0.52-0.89)	1 (ref)	1.24 (1.07-1.44)
Model 3^c^	1 (ref)	0.87 (0.67-1.13)	0.69 (0.53-0.90)	1 (ref)	1.24 (1.07-1.44)
Model 4^d^	1 (ref)	0.87 (0.67-1.13)	0.70 (0.54-0.92)	1 (ref)	1.22 (1.05-1.42)
Model 5^e^	1 (ref)	0.82 (0.63-1.06)	0.70 (0.53-0.91)	1 (ref)	1.22 (1.05-1.43)

ref = referent level; other abbreviations as in Table 1.

a Model 1: Either CRF or CAC, adjusted for age, year of exam, body mass index, smoking, and education, with baseline hazards stratified by sex.

b Model 2: As model 1 but with additional adjustment for glucose, cholesterol, and systolic blood pressure.

c Model 3: As model 2 but with both CRF and CAC; represents the primary adjusted model.

d Model 4: As model 3 but with additional adjustment for antihypertensive and statin therapy.

e Model 5: As model 4 but with additional adjustment for interim stroke/TIA.

f Unadjusted incidence density per 1,000 person-years.

In illness-death models with sequential adjustment for covariates, high CRF was associated with a reduced risk of incident dementia. In contrast, clinically meaningful CAC was associated with an increased risk (Table 2). In the primary adjusted model, which accounted for sociodemographic measures, cardiovascular risk factors, and mutual adjustment for CRF and CAC, participants with high (vs low) CRF had a 31% lower risk of dementia (HR: 0.69; 95% CI: 0.53-0.90), whereas CAC ≥100 AU was associated with a 24% higher risk (HR: 1.24; 95% CI: 1.07-1.44). Additional adjustment for antihypertensive and statin therapy and, subsequently, for interim stroke/TIA as a time-dependent covariate minimally altered these estimates. No evidence of interaction was observed (P for CRF×CAC term = 0.96), indicating independent associations with dementia risk rather than synergistic or antagonistic effects. Consistent with this finding, among participants with very high CAC burden (≥1,000 AU), dementia incidence rates (95% CIs) decreased across fitness categories, from 26.5 (16.0-44.0) to 23.5 (17.7-31.3) to 14.2 (10.7-18.9) per 1,000 person-years in the low-, moderate-, and high-fitness groups, respectively.

**Figure 2:**
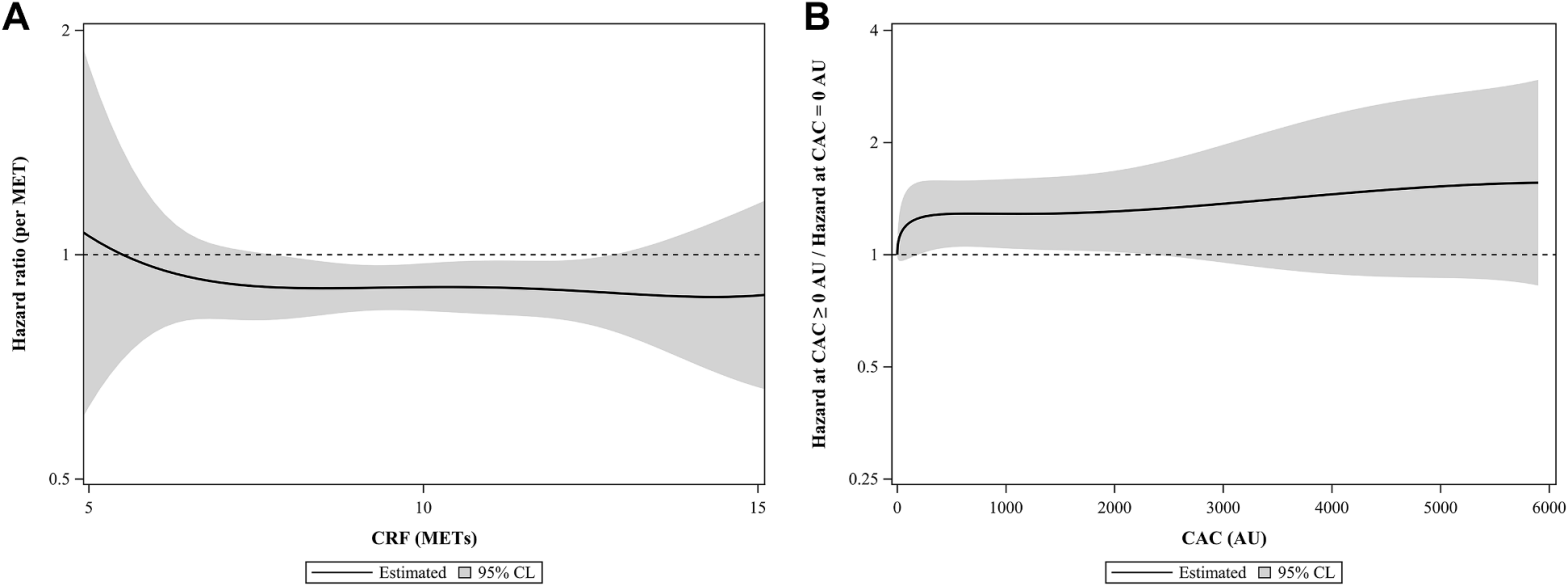
**Nonlinear Analyses of CRF and CAC in Relation to Dementia** Nonlinear analyses illustrating the associations of (A) cardiorespiratory fitness (CRF; left panel) and (B) coronary artery calcium (CAC; right panel) with risk of incident all-cause dementia. Solid lines represent estimated HRs, and shaded areas denote 95% confidence limits. For CRF, HRs are shown per 1-MET increase. For CAC, HRs are shown relative to CAC = 0 AU. Models were estimated using parametric illness-death models with semicompeting risks, with baseline hazards stratified by sex, and adjusted for age, exam year, education, body mass index, current smoking, fasting glucose, total cholesterol, systolic blood pressure, and the alternate exposure (CAC in the CRF model; CRF in the CAC model). Widening CIs at the extremes reflects sparse data in those ranges. AU = Agatston units; CAC = coronary artery calcium; CRF = cardiorespiratory fitness; MET = metabolic equivalent of task.

When CRF and CAC were examined nonlinearly using Chebyshev polynomial expansions, the results were consistent with the categorical classifications (Figure 2). For CRF, dementia risk decreased steadily across most of the observed range, with HRs consistently below 1 from approximately 5 to 15 METs. These findings were concordant with a model treating CRF as a linear variable, which demonstrated a 9% lower risk of dementia per 1-MET higher fitness level (HR: 0.91; 95% CI: 0.87-0.95), after adjustment for age, sex, exam year, BMI, smoking, glucose, cholesterol, systolic blood pressure, and CAC. For CAC, nonlinear analyses showed a graded, approximately monotonic association with dementia risk, with progressively higher HRs across increasing CAC levels and greater uncertainty at the upper extremes of the distribution.

### Apolipoprotein E phenotype by CRF and CAC categories

ApoE genotype data were available for a subset of 1,790 participants. Its distribution across CRF and CAC categories is shown in Table 3. As expected, the ApoE ε3/ε3 variant was the most common across all CRF and CAC categories, accounting for the majority of participants in each stratum. Overall, the distribution of ApoE variants varied only modestly across CRF and CAC categories, with a similar prevalence of ε4 carriers across all strata. Consistent with these observations, analyses treating CAC and CRF as continuous variables revealed no significant differences across ApoE variants (Kruskal-Wallis test: *P* = 0.93 for CRF and *P* = 0.12 for CAC).

**Table 3:** Apolipoprotein E Variants by Cardiorespiratory Fitness and Coronary Artery Calcium (n = 1,790)

ApoE variant	CRF			CAC	
	Low (n = 103)	Moderate (n = 497)	High (n = 1,190)	<100 AU (n = 1,267)	≥100 AU (n = 523)
ε2/ε2, n (%)	2 (1.9)	3 (0.6)	6 (0.5)	9 (0.7)	2 (0.4)
ε2/ε3, n (%)	8 (7.8)	53 (10.7)	141 (11.9)	158 (12.5)	44 (8.4)
ε2/ε4, n (%)	4 (3.9)	13 (2.6)	22 (1.9)	31 (2.5)	8 (1.5)
ε3/ε3, n (%)	59 (57.3)	323 (65.0)	694 (58.3)	738 (58.3)	338 (64.6)
ε3/ε4, n (%)	29 (28.2)	95 (19.1)	300 (25.2)	304 (24.0)	120 (22.9)
ε4/ε4, n (%)	1 (1.0)	10 (2.0)	27 (2.3)	27 (2.1)	11 (2.1)

Abbreviations as in Table 1.

### Sensitivity analyses

Results were robust across multiple sensitivity analyses. First, when CAC was classified into standard categories (0, 1-99, 100-399, 400-999, and ≥1,000 AU), dementia risk generally increased across higher CAC categories. Compared with participants with CAC = 0 AU, the adjusted HRs (95% CIs) were 1.06 (0.87-1.28), 1.21 (0.98-1.50), 1.46 (1.15-1.84), and 1.18 (0.91-1.53), respectively (P for trend = 0.032). Second, inclusion of participants with prevalent CVD at baseline did not materially alter the findings. In this expanded sample, the corresponding HRs (95% CIs) for dementia were 1.25 (1.07-1.45) for CAC ≥100 vs <100 AU and 0.72 (0.55-0.94) for high vs low CRF.

Third, sex-specific analyses showed no evidence that the associations of CRF or CAC with dementia differed by sex (P for CRF×sex term = 0.53; P for CAC×sex term = 0.60). In sex-stratified models, the adjusted HRs (95% CIs) comparing CAC ≥100 vs <100 AU were 1.24 (1.05-1.47) in men and 1.26 (0.92-1.72) in women. The corresponding HRs comparing high vs low CRF were 0.63 (0.46-0.87) in men and 0.81 (0.50-1.32) in women. Overall, these findings support similar associations of CRF and CAC with dementia risk in men and women.

## Discussion

### Principal findings

In a large, well-characterized cohort of predominantly middle-aged adults with objective assessment of CRF and CAC, this study provides one of the first evaluations of their independent and joint associations with later-life dementia risk. High CRF was associated with lower dementia incidence, whereas clinically meaningful CAC (≥100 AU) was associated with a higher dementia risk. These associations remained after adjustment for demographic, behavioral, and cardiometabolic risk factors, including mutual adjustment for CRF and CAC. An additional adjustment for stroke/TIA as an interim event minimally affected both estimates, suggesting that mechanisms beyond overt cerebrovascular events may contribute to these relationships. Continuous analyses were consistent with the categorical findings; dementia risk declined approximately linearly with increasing CRF across most of the observed range, corresponding to an estimated 9% lower risk per 1 MET higher fitness level. Similarly, CAC showed a graded, approximately monotonic association with higher adjusted dementia risk. CAC burden was lower across higher CRF categories; however, no evidence of interaction between CRF and CAC was observed. The absence of interaction suggests that CRF and CAC reflect distinct, independent pathways linking cardiovascular health to brain aging rather than synergistic effects. Descriptive analyses of ApoE showed only modest variation across CRF and CAC categories, suggesting the observed associations are unlikely to be strongly driven by the distribution of ApoE variants. Overall, these findings place objectively measured fitness and subclinical atherosclerosis within a unified framework for understanding midlife cardiovascular contributions to dementia risk (Central Illustration).

**Central Illustration:**
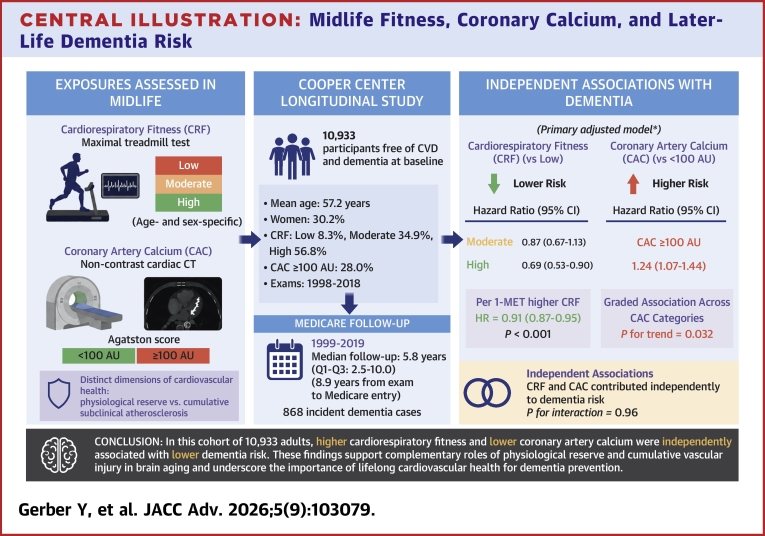
**Midlife Fitness, Coronary Calcium, and Later-Life Dementia Risk** Among 10,933 adults free of cardiovascular disease and dementia at baseline, cardiorespiratory fitness (CRF) and coronary artery calcium (CAC) were assessed in midlife and related to subsequent dementia identified through Medicare surveillance. Higher CRF was associated with lower dementia risk, whereas higher CAC burden was associated with greater risk. Associations remained after adjustment for individual, behavioral, and cardiometabolic risk factors and were consistent in categorical and continuous analyses. No evidence of interaction was observed, indicating that CRF and CAC contribute independently to dementia risk. These findings support complementary roles of physiological reserve and cumulative vascular injury in brain aging and underscore the importance of lifelong cardiovascular health for dementia prevention. ∗Primary adjusted model mutually adjusts for both CRF and CAC and includes: age, sex, year of exam, body mass index, smoking status, education, fasting glucose, total cholesterol, and systolic blood pressure (baseline hazards stratified by sex). AU = Agatston units; CVD = cardiovascular disease; CT = computed tomography; MET = metabolic equivalent of task.

### Dementia epidemiology and prevention

Despite major global efforts, the effectiveness of disease-modifying therapies for the treatment of dementia remains limited.^2^^,^^38^ In this context, growing recognition of the role of vascular health in cognitive aging has reshaped dementia prevention strategies. Observational studies suggest that improved control of cardiovascular risk factors and healthier lifestyle patterns may already be contributing to declines in age-specific dementia incidence in some high-income populations.^39^, ^40^, ^41^ Reflecting this shift, the Lancet Commission on Dementia Prevention has estimated that a substantial proportion of dementia cases may be attributable to modifiable factors, many of which overlap with established cardiovascular risk domains.^1^ Indeed, elimination of key modifiable risk factors (overweight, hypertension, diabetes, cholesterol, smoking, and low education) could reduce dementia incidence by up to 30%.^1^^,^^42^ Together, these observations support a life-course approach to dementia prevention. Our findings extend this literature by jointly examining objective measures of vascular and functional health in mid-to-late midlife and demonstrating independent associations of both CRF and CAC with later-life dementia risk.

### Vascular risk factors for dementia

Vascular risk factors, including hypertension, diabetes, dyslipidemia, smoking, obesity, and physical inactivity, have been associated with an increased risk of cognitive decline and dementia in longitudinal studies.^8^, ^9^, ^10^ These associations often persist after accounting for clinically overt stroke, indicating that vascular contributions to dementia extend beyond major cerebrovascular events. Neuropathological and neuroimaging studies show that subclinical vascular injury is common in older adults and contributes to cognitive impairment. Cerebral small vessel disease, white matter hyperintensities, silent infarcts, and microbleeds have been consistently associated with worse cognitive performance and higher dementia risk.^6^^,^^43^ These vascular lesions frequently coexist with Alzheimer-type pathology, supporting the concept that vascular and neurodegenerative processes jointly influence the clinical expression of dementia.^44^^,^^45^ Vascular risk factors may also contribute to dementia through shared pathophysiological pathways, including chronic hypoperfusion, arterial stiffness, endothelial dysfunction, oxidative stress, and inflammation, which have been implicated in amyloid and tau pathology, neuroinflammation, and neuronal injury.^46^ In this context, cardiovascular imaging biomarkers that integrate lifetime exposure to vascular risk factors, such as CAC, may serve as indicators of cumulative vascular burden relevant to brain aging.^15^^,^^23^^,^^47^ Consistent with this model, our study provides one of the first pieces of evidence that higher CAC was independently associated with increased risk of later-life dementia. Conversely, higher CRF, which reflects better vascular and metabolic health and may mitigate many of these adverse processes, was associated with lower dementia risk. These results underscore the interplay between physiological resilience and cumulative vascular injury in shaping dementia susceptibility.

### Cardiorespiratory fitness and dementia

CRF is an objective measure of integrated cardiovascular, pulmonary, and muscular function. Higher CRF has been consistently associated with lower risk of CVD and mortality.^13^^,^^14^ A growing body of evidence also supports an inverse association between CRF and dementia risk. Several prospective cohort studies, including CCLS, have shown that higher midlife CRF (assessed by maximal exercise testing) is associated with a lower incidence of all-cause dementia decades later.^17^^,^^19^, ^20^, ^21^ Unlike earlier CCLS analyses, the present study draws on more recent clinic examinations and Medicare follow-up, thereby allowing improved temporal alignment between exposure and outcome. Large studies among U.S. veterans have demonstrated a graded inverse association, with approximately 8% to 10% lower dementia risk per 1-MET higher fitness level.^48^^,^^49^ Very recently, a study of over 60,000 dementia-free adults in the UK Biobank, followed for up to 12 years, reported that higher CRF (assessed via submaximal exercise test) was associated with a lower long-term dementia risk, with evidence that high fitness attenuated dementia risk even among individuals with moderate or high genetic susceptibility.^18^ Findings from the HUNT (Trøndelag Health Study) similarly suggested that maintaining or improving CRF (estimated using a nonexercise prediction model) is associated with a lower incidence of dementia.^50^ Consistent with this literature, our study, with up to 21 years of Medicare follow-up, demonstrates that high midlife CRF is associated with lower dementia incidence after extensive adjustment, including cardiovascular risk factors, CAC, antihypertensive and statin therapy, and interim stroke/TIA. Continuous analyses further support a largely inverse linear association across most of the observed fitness range.

### Coronary artery calcium and dementia

CAC is a marker of subclinical atherosclerosis that reflects the cumulative impact of measured and unmeasured cardiovascular risk factors on the arterial wall.^15^ CAC has demonstrated strong, graded associations with future CVD events across diverse populations, adding predictive value beyond traditional risk factor assessment.^14^, ^15^, ^16^^,^^25^ Increasing interest has therefore focused on whether CAC may also capture vascular processes relevant to brain aging and dementia risk. Evidence linking CAC to incident dementia, however, has been more limited and heterogeneous.^24^ In the Multi-Ethnic Study of Atherosclerosis, higher CAC burden was associated with an increased risk of incident dementia (271 cases) after adjustment for traditional cardiovascular risk factors and ApoE. The association was attenuated with further adjustment for stroke and, particularly, CVD as time-dependent interim events.^23^ In contrast, findings from the Rotterdam Study indicated that larger calcification volumes in the aortic arch, extracranial carotid arteries, and intracranial carotid arteries, but not in the coronary arteries, were associated with higher dementia risk.^22^ In this context, a prospective study of community-dwelling older women demonstrated that higher abdominal aortic calcification, assessed on lateral spine images, was associated with a substantially higher risk of late-life dementia hospitalization and mortality over long-term follow-up, independent of cardiovascular risk factors and ApoE.^51^ Although abdominal aortic calcification and CAC are correlated and reflect shared systemic atherosclerosis, they are not interchangeable and may capture distinct aspects of vascular disease burden relevant to cognitive outcomes. Our findings support an association between CAC and later-life dementia risk that is independent of traditional cardiovascular risk factors, CRF, antihypertensive and statin therapy, and interim stroke/TIA. Together with prior studies of vascular calcification in other arterial beds, these results suggest that CAC represents one manifestation of systemic vascular disease relevant to cognitive aging.

### Strengths and limitations

Key strengths of this study include the objective measurement of CRF via maximal treadmill testing, standardized assessment of CAC, and long-term follow-up from midlife into older age in a large, well-characterized cohort. The application of illness-death models allowed for appropriate accounting for the competing risk of death, and linkage to Medicare claims enabled ascertainment of outcomes in later life. To our knowledge, this is among the first cohort studies to jointly evaluate CRF and CAC in relation to dementia risk. Findings support a model in which physiological reserve and cumulative vascular injury contribute independently to neurodegeneration and cognitive decline.

Several limitations should be considered when interpreting these findings. Dementia was ascertained using Medicare administrative claims rather than clinical adjudication, which may result in misclassification, although this approach has demonstrated reasonable validity in prior studies.^35^ Differential health care utilization may also have influenced ascertainment. Participants with greater CAC burden may have had more frequent health care encounters and, therefore, more opportunities to receive dementia-related diagnostic codes. Because all CCLS participants underwent preventive health evaluations and generally had access to health care, differences in diagnostic ascertainment may be less pronounced than in more heterogeneous populations. Nevertheless, some degree of differential detection cannot be excluded. In addition, we may not have captured all dementia cases because surveillance began after the clinic visit. In this cohort, the median interval between the clinic visit and Medicare surveillance was 8.9 years. Because participants had to survive and remain free of dementia until surveillance began, survivorship selection may have occurred, potentially retaining individuals with more favorable CRF and CAC profiles. The direction and magnitude of any resulting bias are difficult to predict. Nevertheless, dementia before age 65 is uncommon, and death before Medicare eligibility was rare in this cohort. Indeed, Medicare surveillance began nearly 2 decades before the estimated mean age of dementia onset in the United States (83.7 years),^52^ reducing the likelihood of substantial outcome misclassification. The interval between exposure assessment and outcome surveillance also reduces concerns about reverse causation. Our use of all-cause dementia as the primary outcome may have further diluted associations with specific dementia subtypes. Neuropathological studies indicate that multiple cerebral pathologies commonly coexist with advancing age, suggesting that associations with any single etiology may be attenuated when dementia is considered as a composite outcome.^53^ ApoE genotype, considered a major genetic risk factor for Alzheimer disease,^6^^,^^34^ was available only for a subset of the sample. However, in Multi-Ethnic Study of Atherosclerosis, adjustment for the ApoE ε4 allele did not materially alter the association between CAC and dementia.^23^ Consistent with this, descriptive analyses in the subsample of CCLS participants showed only modest variation in ApoE variants across CRF and CAC categories. The cohort is predominantly White, well educated, and relatively healthy, which may limit generalizability. CRF was estimated from performance on a symptom-limited maximal treadmill test rather than directly measured by cardiopulmonary exercise testing; however, the modified Balke protocol has been extensively validated and is highly correlated with measured VO_2_max. In addition, CRF and CAC were assessed once in this analysis, precluding evaluation of trajectories, which may provide additional insight into dementia risk and warrant investigation in future studies. Although exposures were assessed primarily in middle-aged adults (mean age 57 years), some participants were older at baseline; therefore, our findings may not strictly reflect early midlife exposures.

## Conclusions

In this large cohort of U.S. adults without symptomatic CVD, higher CRF and lower CAC burden from midlife to early older adulthood were independently associated with a reduced risk of later-life dementia, supporting their complementary use in risk stratification and prevention strategies for brain health. These findings highlight distinct, nonoverlapping dimensions of cardiovascular risk relevant to dementia and underscore the importance of maintaining cardiovascular health across the life course.

Perspectives **COMPETENCY IN MEDICAL KNOWLEDGE:** Midlife CRF and subclinical coronary artery disease, quantified via CAC, provide independent, complementary, and nonoverlapping risk stratification for later-life dementia. A higher level of objective functional fitness in midlife is strongly associated with a reduced risk of incident all-cause dementia, whereas a clinically meaningful burden of subclinical coronary atherosclerosis (≥100 AU) is associated with an elevated risk.

Because no statistical interaction has been observed between these 2 markers, they likely reflect distinct, parallel pathways linking lifetime cardiovascular health to brain aging. Crucially, these independent associations remain robust even after accounting for interim stroke or TIA, demonstrating that subclinical systemic vascular injury contributes to dementia pathogenesis through mechanisms that extend far beyond overt, clinical cerebrovascular events.

**TRANSLATIONAL OUTLOOK:** Future longitudinal cohort studies should focus on evaluating trajectories and dynamic changes in CRF and CAC over time, rather than single-point exposures, to better understand how progressive changes in cardiovascular and functional health influence cognitive decline. Furthermore, because the current findings are derived from a predominantly White, highly educated, and relatively healthy cohort, additional research in racially, ethnically, and socioeconomically diverse populations is required to confirm generalizability. Lastly, investigating the specific relationships between these midlife cardiovascular biomarkers and distinct neuropathological dementia subtypes (such as Alzheimer disease vs vascular dementia) will help delineate the exact vascular and neurodegenerative mechanisms at play.

## Funding support and author disclosures

Prof. Gerber is supported in part by the Lilian and Marcel Pollak Chair in Biological Anthropology at Tel Aviv University, Israel. All other authors have reported that they have no relationships relevant to the contents of this paper to disclose.
